# Atopic dermatitis prevention in children following maternal probiotic supplementation does not appear to be mediated by breast milk TSLP or TGF-β

**DOI:** 10.1186/s13601-016-0119-6

**Published:** 2016-07-22

**Authors:** Melanie Rae Simpson, Anne Dorthea Bjerkenes Rø, Øystein Grimstad, Roar Johnsen, Ola Storrø, Torbjørn Øien

**Affiliations:** Department of Public Health and General Practice, Norwegian University of Science and Technology, Trondheim, Norway; Department of Immunology and Transfusion Medicine, St Olavs Hospital, Trondheim University Hospital, Trondheim, Norway; Department of Dermatology, University Hospital of North Norway, Tromsö, Norway; Det medisinske fakultet, Instiutt for samfunnsmedisin, MTFS, NTNU, Postboks 8905, 7491 Trondheim, Norway

## Abstract

**Background:**

The Probiotics in Prevention of Allergy among Children in Trondheim (ProPACT) study, a randomised, placebo controlled trial, demonstrated that maternal supplementation with probiotic milk reduced the incidence of atopic dermatitis (AD) in infancy. The mechanisms behind this effect are incompletely understood and breast milk cytokines have been postulated as possible mediating factors. In this study we aimed to assess whether breast milk TLSP and TGF-β are affected by a maternal probiotic supplementation regime, and their contribution to the preventive effect of this regime on AD in the offspring.

**Methods:**

TSLP and TGF-β isoforms (TGF-β_1_, TGF-β_2_ and TGF-β_3_) were measured using ELISA and multiplex assays, respectively, in breast milk samples collected at 10 days and 3 months postpartum from women participating in the ProPACT trial (n = 259). The natural indirect and direct effects of maternal probiotics on AD, due to changes in breast milk cytokines, were estimated using causal mediation techniques.

**Results:**

Probiotic supplementation tend to lead to high levels of breast milk TSLP at 10 days postpartum (p = 0.062), but this change did not contribute to the prevention of AD according to the mediation analysis. Probiotics had no apparent effect on TSLP at 3 months or TGF-βs at either time points. Thus, these are unlikely to be mediators of the effect of maternal probiotics on AD in offspring.

**Conclusions:**

Whilst maternal probiotic supplementation resulted in higher breast milk concentrations of TLSP at 10 days postpartum, this does not appear to be a mechanism for prevention of AD by maternal probiotics.

*Trial registration* The original trial protocol is registered in ClinicalTrials.gov (identifier NCT00159523)

## Background

Perinatal supplementation with probiotics has been shown to reduce the incidence of atopic dermatitis (AD) in infancy [1, 2]. Our own study, the Probiotics in the Prevention of Allergies among Children in Trondheim (ProPACT) trial, demonstrated a 40 % reduction in the development of AD following maternal probiotic supplementation [3]. However, the biological mechanisms behind this effect are incompletely understood. Using samples taken during the ProPACT trial, we have previously reported that the maternal intestinal microbiota is modified by probiotic supplementation, and that children born to mothers who received the probiotics have a higher abundance of *Lactobacillus rhamnosus GG* (LGG) which persists up until 3 months of age [4]. Whilst the transfer of such probiotic bacteria from mother to child may prevent AD, alterations in various components of breast milk have also been postulated as possible mediating factors.

In addition to being a source of nutrition and hydration for newborn infants, breast milk contains a number of immunologically active cells and molecules, such as immunoglobulins, lactoferrins, growth factors and cytokines [5]. Among the cytokines found in breast milk are thymic stromal lymphopoietin (TSLP) and the transforming growth factor-β (TGF-β) cytokines. TSLP has been implicated in the establishment and maintenance of T helper type 2 (Th2) responses, and thus also in defence against helminthic infections and in the pathogenesis of allergy related diseases [6]. Genetic variants of TSLP have been associated with AD and asthma [7] and high levels of epidermal TSLP precede the clinical presentation of childhood AD [8], suggesting that TSLP may be particularly important in the establishment of AD. Other studies have demonstrated that TSLP activates skin dendritic cells promoting a Th2 response and interacts directly with skin-homing Th2 cells to enhance interleukin-4 (IL-4) production which is thought to contribute to the maintenance of inflammation in chronic AD [9, 10]. Consistent with these studies, higher concentrations of TSLP have been reported in both acute and chronic AD lesions [6]. Murine models have shown that over-expression of TSLP in keratinocytes induces an AD-like skin disease and can predispose to allergic airway inflammation after intranasal challenge. Thus, TSLP may also be involved in the progression to other allergy related diseases, a process often referred to as the atopic march [6]. However, the biological effects of breast milk TSLP for the mother and child are unknown [11], and this cytokine is previously unstudied within the context of maternal probiotic supplementation. Neither has the effect of maternal atopy on breast milk TSLP been investigated, nor the association between breast milk TSLP and the development of AD. In contrast to TSLP, the human isoforms of TGF-β (TGF-β_1_, TGF-β_2_ and TGF-β_3_) are primarily implicated in inhibition of allergic inflammation through a wide range of immunoregulatory effects [12]. All three isoforms of TGF-β are found in breast milk. Studies investigating the effect of probiotic supplementation on breast milk TGF-β concentrations and or their association with later allergy related disease in offspring, have produced conflicting results [13–18].

Both TSLP and TGF-βs appear to have important roles in acute and chronic phases of allergy related disease, which makes them interesting as potential mediators of this preventive effect. The aims of the current study were to: (a) determine if perinatal maternal probiotic supplementation alters the concentration of TLSP, TGF-β_1_, TGF-β_2_ or TGF-β_3_ in breast milk at 10 days and 3 months postpartum and (b) investigate if these breast milk cytokines contribute to the preventative effect of maternal probiotic supplementation on the development of AD at 2 years of age through causal mediation analysis.

## Methods

### Participant recruitment and sample collection

The ProPACT trial enrolled 415 pregnant women who were randomised to receive 250 mL per day of probiotic or placebo milk from 36 weeks gestation until 3 months postpartum [3]. The probiotic milk contained 5 × 10^10^ colony-forming units (CFUs) of *Lactobacillus rhamnosus GG* (LGG) and *Bifidobacterium animalis* subsp. *lactis* Bb-12 (Bb-12) and 5 × 10^9^ CFU of *L. acidophilus* La-5 (La-5) per serving. The placebo was a fermented skim milk, pasteurised after fermentation and contained no probiotic bacteria. Participants completed lifestyle questionnaires at baseline (~30–36 weeks gestation), 6 weeks, 1 and 2 years postpartum and child health questionnaires at 1 and 2 years of age. The outcome of interest in the current study was the development of AD by 2 years of age as diagnosed by a paediatrician using the United Kingdom (UK) Working Party Diagnostic criteria [19]. Detailed descriptions of the ProPACT study design and clinical outcomes have been published previously [3, 20].

Breast milk samples were collected into a sterile container at 10 days and 3 months postpartum and stored in the participant’s home freezer until transported to the laboratory in Styrofoam containers to prevent thawing. Samples were subsequently stored at −80 °C until analysis. All mother-infant pairs that attended the 2 year clinical follow-up and submitted at least one breast milk sample were eligible for inclusion in this study.

#### Cytokine quantification

Breast milk samples were thawed and centrifuged (16,100*g*, 10 min, 4 °C) to remove the lipids, cells and debris. The aqueous portion was used for subsequent cytokine quantification. TSLP concentrations were measured using a human TLSP ELISA kit (R&D Systems, Minneapolis, USA) according to the manufacturer’s instructions. The standards and an internal control were conducted in triplicate. The intra-assay coefficient of variation (CV) was <13.5 % and inter-assay CV was 22 %. A large proportion of the samples had TSLP concentrations outside of the detection limits of the assay, both above and below. We have therefore categorised the TSLP concentrations into the following 4 categories: below detection, low detectable, high detectable and above detection. The internal control sample was chosen as the cut-off between the low and high detectable categories, because it was approximately the median concentration and maximised the inter-assay comparability. For the purpose of mediation analysis TSLP was categorised into a binary variable above and below the internal control.

TGF beta concentrations were measured using a multiplex assay (Bio-Plex Pro TGF-β assay, Bio-Rad Laboratories, Oslo, Norway) following the manufacturer’s instructions which included an actvation step. Prior to analysis, 100 µL of the aqueous portion of breast milk was activated using 20 µL 1 N HCl for 10 min at room temperature, followed by neutralisation using 20 µL 1 N NaOH with 0.5 M HEPES. The standard and two internal control samples were conducted in duplicate. Concentrations were calculated using standard curves and scaled by 1.4 due to dilution in the activation-neutralisation process. The intra-assay CVs were <13.6, <13.4 and <14.6 % and the inter-assay CV were <9.9, <5.9 and <14.3 % for TGF-β_1_, TGF-β_2_ and TGF-β_3_, respectively.

### Statistical analysis

As described, TSLP concentrations were categorised into a four groups, and the effect of probiotics was assessed using ordinal logistic regression. The distributions of TGF-βs were right-skewed and concentrations are reported as medians and interquartile ranges. Wilcoxon matched-pairs signed-rank test was used to compare the concentration at 10 days and 3 months for the 243 women with TGF-β isoforms measured at both time points. The effect of probiotic supplementation on TGF-β concentrations was assessed using linear regression on log-transformed concentrations. Maternal atopy, maternal smoking during the first year of life and the presence of older siblings were considered to be potential moderators of the effect of probiotics on cytokine concentrations. The effect of probiotics on the breast milk cytokines were therefore also assessed in alternate regression models which included these covariates.

Causal mediation analysis was performed using the user written command, *paramed,* for breast milk cytokines which were found to be altered by probiotic supplementation. This analysis estimated the natural indirect effect (NIE) and natural direct effect (NDE) of maternal probiotic supplementation on the development of AD in offspring, mediated through breast milk cytokine concentrations, using counterfactual definitions of these effects and a log-binomial regression model for AD, given AD is not rare [21–23]. In this study, the NIE represents the effect of the probiotic regime on the development of AD which can be attributed to its effect on the breast milk cytokine. The NDE represents an estimation of what the relative risk (RR) of developing AD after maternal probiotic supplementation would have been, had there been no effect on the concentration of the breast milk cytokine. The *paramed* command does not currently support models with ordinal mediator variables and TSLP concentrations were therefore dichotomised for mediation analysis. Whilst the randomised design ensures that the effect of probiotics on breast milk cytokines and on AD can be estimated without confounding, the relationship between cytokine concentration in breast milk and the development of AD is not randomised. As such, the estimation of NIE and NDE may be confounded by factors which influence both breast milk cytokine concentrations and the risk of developing AD in offspring. The previously mentioned covariates were therefore included in the mediation analysis as potential confounders of the relationship between breast milk composition and AD. All statistical analyses were performed using Stata IC release 13 (StataCorp, College Station, Texas).

## Results

### Participants

**Fig. 1:**
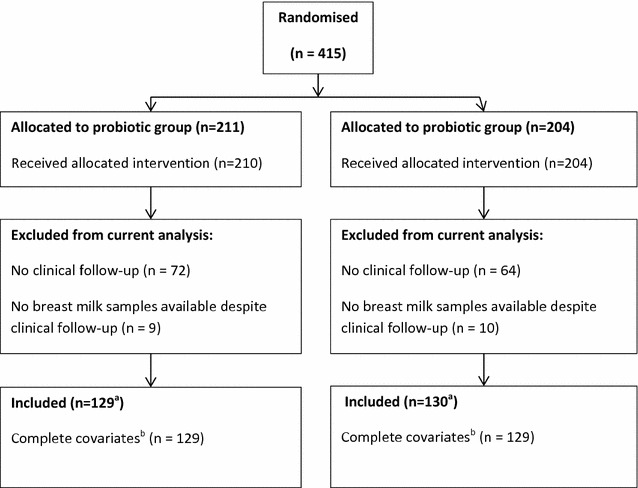
Flow diagram of participants in the ProPACT study and cytokine analysis of breast milk samples. ^a^The provided values represent the number of eligible women who had provided at least one breast milk sample. The precise number of samples analysed for each cytokine at each time point varies and is provided in association with the relevant results. ^b^Complete covariates additionally required information on maternal atopy, maternal smoking and the presence of older siblings

Two hundred and fifty-nine mother-infant pairs were included in this analysis, 129 from the probiotic group and 130 from the placebo group (Fig. 1). This subgroup of participants is representative of the original study population with respect to distribution of baseline characteristics and allergy related disease outcomes [3]. Compliance was high and equivalent in both groups. At baseline, the probiotic group contained more male children and at 2 years of age there were fewer cases of AD (Table 1). In terms of maternal characteristics which have been previously suggested to influence breast milk cytokine concentration, fewer women in the probiotic group had a personal history of atopy (Table 1). There were also fewer women smoking at 6 weeks postpartum in the probiotic group, although the overall prevalence of smoking was very low (8 of 259, 3.2 %).

**Table 1:** Baseline characteristics of participating families and allergy related disease in the children at 2 years

Characteristics	Probiotic		Placebo	
	n		n	
Age, mother, yrs mean (SD)	129	30.5 (3.9)	130	30.3 (4.1)
Sex (male), child, n (%)	129	67 (51.9)	130	53 (40.8)
Siblings, n (%)	129	60 (46.5)	130	54 (41.5)
Atopy in family, n (%)	129	91 (70.5)	130	96 (73.9)
Maternal atopy, n (%)	129	58 (45.0)	129	68 (52.7)
Maternal smoking^a^, n (%)	129	9 (7.0)	130	11 (8.5)
Compliant^b^, n(%)	125	113 (90.4)	128	115 (89.8)

Allergy related disease at 2 years	n	n (%)	n	n (%)	p-value^c^
Atopic dermatitis, n (%)	129	29 (22.5)	130	45 (34.6)	0.031
Asthma, n (%)	129	7 (5.4)	130	12 (9.2)	0.240
Allergic rhinoconjunctivits, n (%)	125	1 (0.8)	129	0 (0.0)	0.492
Sensitisation^d^, n (%)	122	19 (15.6)	124	15 (12.1)	0.429

^a^Maternal smoking reported during pregnancy, 6 weeks or 12 months postpartum; ^b ^Compliance with the study protocol was defined by consumption of the study milk on at least 50 % of days from 36 weeks gestation to 12 postpartum, no consumption of other products with probiotics and at least partial breastfeeding until 3 months postpartum; ^c^ p-value calculated using χ^2^-test, except for allergic rhinoconjunctivits where a Fisher’s exact *p* value is reported; ^d^ Allergic sensitisation defined as positive skin prick test (wheal ≥ 3 mm) and or positive sIgE (≥ 0.35 kUL^−1^)

### Thymic stromal lymphopoietin

**Fig. 2:**
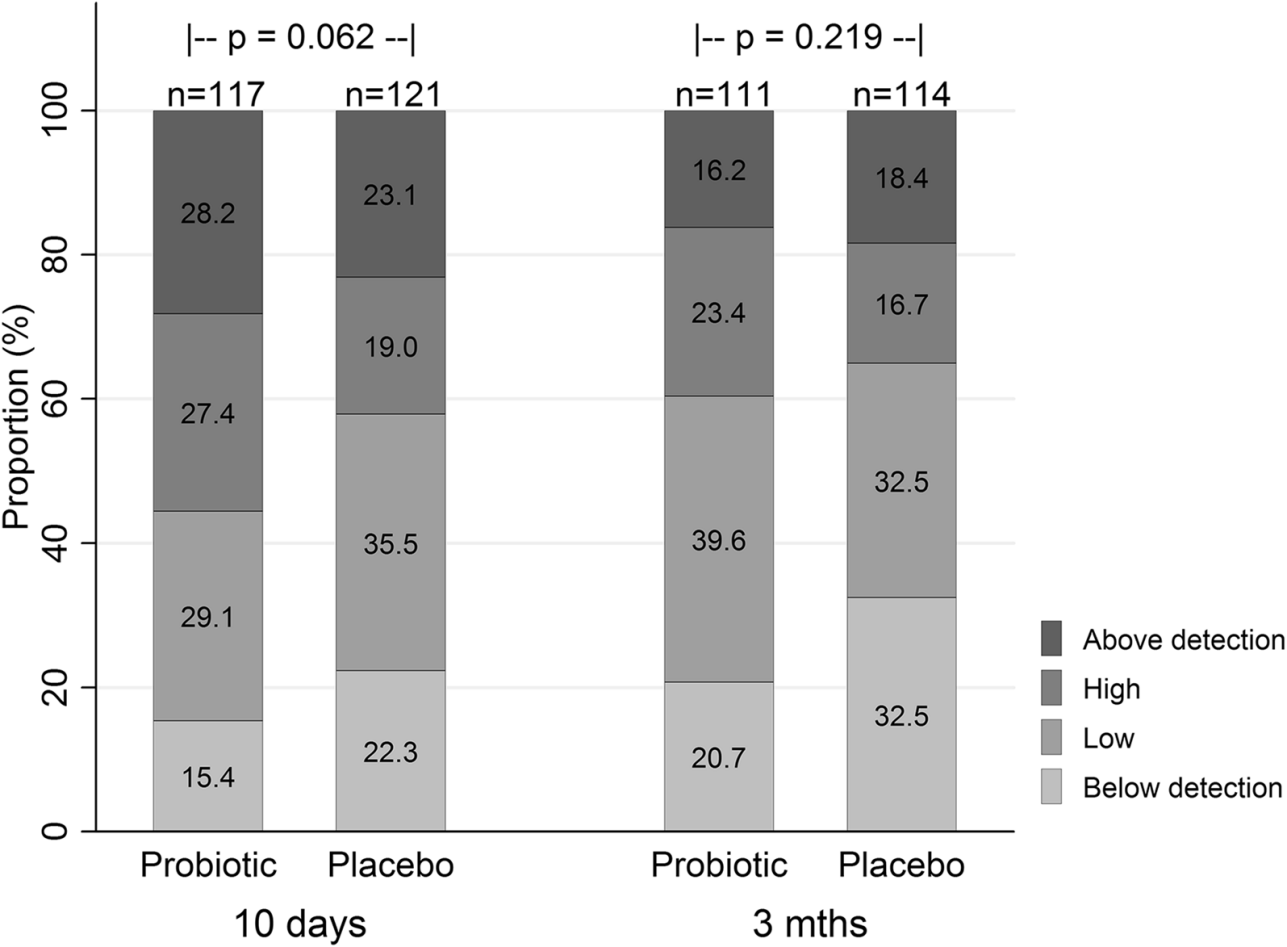
Breast milk TSLP concentrations at 10 days and 3 months in the probiotic and placebo groups. Proportion of breast milk samples with categorised TSLP concentrations at 10 days and 3 months postpartum in the placebo and probiotic group. Percentages are provided within the *bars*. Overall, higher concentrations of TSLP were measured significantly more often in samples collected at 10 days postpartum (p < 0.001 from ordinal logistic regression clustered by individual). Also on subgroup analysis, TSLP concentrations were significantly higher at 10 days in both the probiotic group (p < 0.001) and placebo group (p = 0.005)

A greater proportion of breast milk samples taken 10 days postpartum had high concentrations of TLSP compared with the samples taken at 3 months (Fig. 2). The concentration of TSLP was below the lower limit of detection (31.3 pg/mL) in 18.9 % and 26.7 % of samples collected at 10 day and 3 months postpartum, respectively, and above the limit of detection (2000 pg/mL) in 25.6 % and 17.3 %, respectively. Ordered logistic regression revealed a borderline non-significant effect of probiotics on TLSP concentration (OR 1.55 95 % CI 0.98–2.45, p = 0.062) at 10 days postpartum which was not sustained at 3 months postpartum (OR 1.35 95 % CI 0.84–2.16, p = 0.219). This effect was not significantly altered when adjusting individually or in combination for maternal atopy, maternal smoking or older siblings. The effect of probiotics on breast milk TSLP concentration at 10 days postpartum was marginally enhanced when considering only women compliant with the study protocol (n = 209, OR 1.74, 95 % CI 1.06–2.84, p = 0.028). We did not find evidence of an association between maternal atopy, maternal smoking or the presence of older siblings and TSLP in univariate analysis.

### Transforming growth factor β

**Fig. 3:**
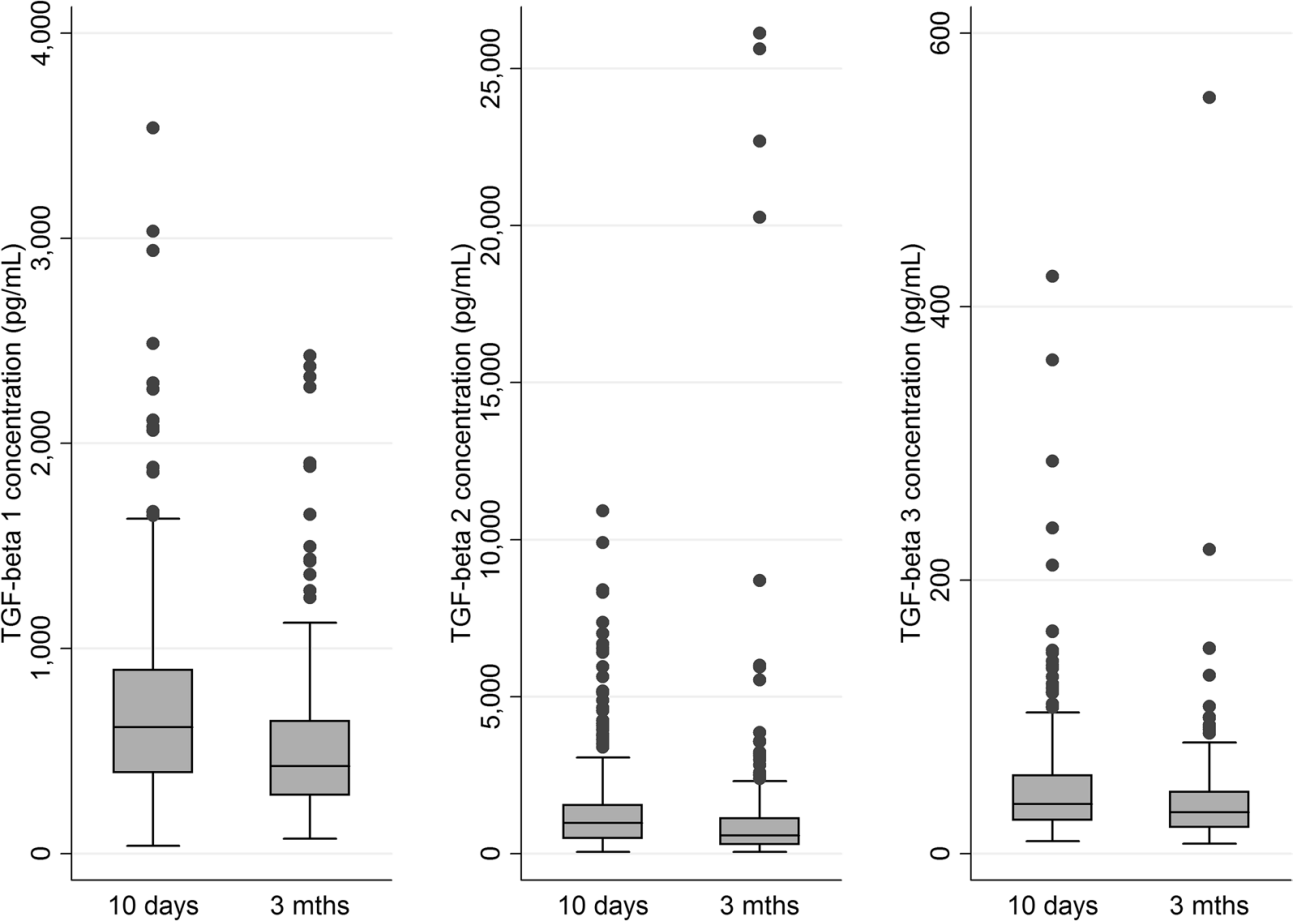
Concentrations of TGF-β1, 2 and 3 at 10 days and 3 months. All three isoforms of TGF-β are found at statistically significantly lower levels at 3 months postpartum compared to 10 days postpartum (p < 0.001 for all isoforms using Mann–Whitney U-test)

All three TGF-β isoforms were detectable in all breast milk samples, with the exception of one sample collected at 3 months postpartum which had undetectable TGF-β_3_. Similar to TSLP, the concentrations of TGF-β_1_, TGF-β_2_ and TGF-β_3_ were higher at 10 days postpartum compared with 3 months postpartum (Fig. 3). Probiotic supplementation had no observed effect on the concentration of any of the TGF-β subtypes at either time point (Table 2). This lack of effect was not substantially altered when adjusting for potential moderators or when considering the subgroup of women compliant with the study protocol (n = 219–224).

**Table 2:** Breast milk TGF-β concentrations in the probiotic and placebo groups at 10 days and 3 months postpartum

	Days	Treatment				p^a^
		Probiotic		Placebo		
		n	Median (IQR) (pg/mL)	n	Median (IQR) (pg/mL)	
TGF-β_1_	10	128	616.9 (407.5–950.4)	127	617.3 (369.7–853.9)	0.34 0.33
	90	123	438.5 (308.0–661.1)	124	417.4 (269.3–641.8)	0.27 0.23
TGF-β_2_	10	128	909.8 (451.6–1653.8)	127	999.4 (519.0–1517.1)	0.90 0.83
	90	123	599.6 (311.0–1055.1)	124	532.5 (285.9–1144.2)	0.76 0.69
TGF-β_3_	10	128	37.8 (25.3–58.7)	127	35.8 (22.9–57.2)	0.25 0.33
	90	123	29.9 (21.0–42.7)	123	30.8 (18.8–48.7)	0.95 0.86

^a^The upper p-value provided is from the regression analysis of the influence of probiotic ingestion on the log transformed TGF beta concentration by, whilst the lower represents the p-value calculated for the effect of treatment allocation after adjusting for maternal atopy, maternal smoking and presence of siblings

### Breast milk cytokines as potential mediators of preventative effect on atopic dermatitis

**Fig. 4:**
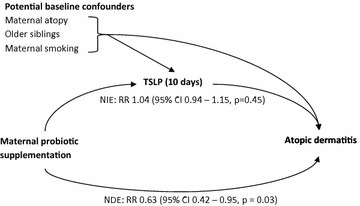
Hypothetical mediation analysis and estimated natural indirect effect (NIE) and natural direct effect (NDE) of maternal probiotic supplementation. The NIE is an estimate of the effect of maternal probiotic ingestion on the development of AD in offspring mediated by increased TSLP levels in breast milk 10 days postpartum. The NDE is an estimate of the effect of maternal probiotic ingestion on the development of AD not mediated through changes in breast milk TSLP concentration 10 days postpartum. This analysis suggests that TSLP does not significantly contribute to, or oppose, the preventative effect of maternal perinatal probiotic supplementation on the development of atopic dermatitis

For the purposes of mediation analysis, TSLP concentrations at 10 days were dichotomised. Consistent with the ordinal logistic regression analysis, standard logistic regression on the dichotomised TSLP values also suggested that perinatal probiotic supplementation increased the odds of a high TSLP concentration at 10 days (OR 1.72, 95 % CI 1.03–2.87, p = 0.039). However, mediation analysis suggested that the effect of probiotics on TSLP did not result in a significant reduction, or increase, in the risk of developing AD [RR^NIE^ 1.04 (95 % CI 0.94–1.15, p = 0.45)], Fig. 4). Breast milk TSLP concentrations at 3 months postpartum and the TGF-β cytokines at both 10 days and 3 months postpartum were not demonstrably affected by probiotic supplementation, indicating that changes in these breast milk cytokines are unlikely to be responsible for the reduced risk of developing AD observed after maternal probiotic supplementation. As such, no estimation of NIE and NDE was conducted for these. Furthermore, none of the cytokines, including TSLP at 10 days, were found to be significantly associated with the development of AD when assessed independently of treatment allocation.

## Discussion

Thymic stromal lymphopoietin was the only cytokine with a tendency to be affected by maternal probiotic supplementation, resulting in higher concentrations of TSLP at 10 days postpartum. Causal mediation analysis indicated that the higher concentrations of TSLP at 10 days did not appear to contribute to the beneficial effect of probiotics on the development of AD in offspring. TSLP concentrations at 3 months postpartum and TGF-βs at both time points were not affected by probiotic supplementation. As such, these breast milk cytokines are unlikely to be mediators of the beneficial effect of probiotics.

This study provides the first report of the influence of a maternal probiotic supplementation on breast milk TLSP concentrations and investigates its contribution to the prevention of AD. In experimental and observational studies, TSLP has been associated with promotion of a Th2 and allergic type inflammation, particularly in the lung and skin [6, 24]. Therefore, the finding that probiotic supplementation increased the breast milk TSLP concentrations was contrary to what one might have expected. At the same time, high breast milk TSLP may not be as detrimental in the intestinal system where it is described as having a more regulatory function [24, 25]. TSLP released by intestinal epithelial cells in response to commensal bacteria has been shown to promote tolerogenic properties in dendritic cells and macrophages which then produce IL-10 and retinoic acid and promote regulatory T helper (Treg) cell differentiation [25]. Thus the increased TSLP seen in the probiotic group may encourage intestinal immune homeostasis, which in turn is theoretically beneficial for the development of the neonatal immune system and prevention of AD. However, the current data does not suggest that breast milk TSLP contributes to the preventative effect of probiotics on AD, nor does it appear to be associated with the development of AD. It is unclear if the observed lack of mediating effect is due to a loss of information and power due to dichotomisation of TSLP concentration, TLSP degradation prior to reaching the intestines, or a more complex interplay between TSLP, the intestinal microbiota and the immune system. Further studies are required to confirm these findings.

The major strength of this study is that the randomised placebo controlled, double blind design of the ProPACT trial allows an unconfounded assessment of the effect of the probiotic regime on the selected breast milk cytokines. Furthermore, through causal mediation analysis techniques, we are able to estimate if and to what extent the prevention of AD is due to changes in TSLP. The randomised design also means that two of four assumptions required for this mediation analysis [21] are automatically satisfied, namely that there is no residual confounding of the effect of probiotics supplementation on either AD or breast milk TSLP. The other two required assumptions are that there is no unmeasured confounding of the relationship between breast milk TSLP and AD and that there is no confounder of the mediator-outcome relationship that is affected by treatment allocation. Whilst these cannot be excluded, we believe we have measured and included all variables which are likely to affect both breast milk cytokines and AD and that probiotics do not have a strong effect on any other confounders of the relationship between these variables. Another strength of this study is that it is the largest to investigate TSLP concentrations in breast milk and the only one to consider infant outcomes and maternal atopy status. The only other study to report breast milk TSLP concentrations involved 44 women who submitted samples at one of two time points [11]. Whilst they also found that mature milk tended to have lower TSLP concentrations, they reported concentrations which all fell within a relatively narrow range. Our results suggest that there may be a much greater variability between individuals than this small study managed to detect.

One of the limitations of this study is that the breast milk samples were stored between 6 and 8 years before analysis. This has potentially affected the measured concentrations of TSLP and TGF-βs, as has been previously reported for TGF-β_1_ [26]. However, there was no difference in the average length of storage between the treatment groups and adjusting for length of storage did not significantly alter the estimated effect of probiotic supplementation on any of the measured cytokines (data not shown). Another limitation is that the time of sample collection was not standardised with respect to time of day and whether fore- or hindmilk was collected. Although diurnal and fore-/hindmilk variation has not been specifically investigated for TSLP and TGF-βs, previous studies suggest that diurnal variation exists in some breast milk cytokines [27] and that the quantity of total protein in the aqueous portion of fore- and hindmilk is reasonably constant [28]. Whilst these factors may have influenced the cytokine concentrations, samples from the probiotic and placebo groups are presumably equally affected and we do not believe they have significantly affected the conclusions of this study. An underlying uncertainty surrounding the measure of breast milk cytokines is another limitation to this study. Previous studies of breast milk TGF-β and other cytokines have produced widely varying concentration measurements. Whilst some of this variability may be explained by factors such as diurnal and fore-/hindmilk variations, ethnicity, maternal atopy status and time point postpartum, it is likely that sample storage, preparation, analysis methods and inter-laboratory differences play a major role in the measured concentrations. As such, it is difficult to compare results between studies, particularly when these variables are inconsistently reported. In studies comparing two exposure groups, be that probiotic versus placebo or maternal atopy versus no atopy, it is reasonable to assume that technical variability between the groups are minimised. Thus, comparing groups within each study is valid and more generally the relative relationship between these groups can be roughly compared across studies.

Five previous studies have reported breast milk TGF-β_1_ and or TGF-β_2_ concentrations from similar randomised trials of probiotic supplementation. Each of these studies found a statistically significant effect of probiotics on at least one TGF-β isoform in colostrum samples and no effect on concentrations in mature milk. However, they provide conflicting evidence as to whether probiotics increase [14, 15] or decrease [16–18] colostrum TGF-β concentrations. The current study also found no effect of probiotic supplementation on TGF-β concentrations in mature milk, and provides no evidence to suggest either increased or decreased concentrations in breast milk at 10 days postpartum, a time when the milk is sometimes referred as transition milk before colostrum become mature milk. In addition to the previously mentioned sources of variability, the differing results may also reflect a probiotic strain specific effect as none of these studies used the same probiotic species in the same dose or combinations. In terms of methodological differences, it is interesting to note that the two studies reporting reduced TGF-β_2_ after probiotic supplementation also reported the manufacturer recommended acid activation of the breast milk samples prior to measurement which means that the reported concentrations represent the entire pool of TGF-β which is naturally found in both a latent and active form. On the other hand, all three studies reporting higher TGF-β_1_ or TGF-β_2_ do not mention this activation step in their methods. One of these studies, however, used an alternate activation procedure which is thought to additionally release lipid bound TGF-β [17, 29]. All other studies have removed the lipid fraction prior to cytokine analysis. If the conflicting reports of positive or negative associations with probiotics are a reflection of a common effect of probiotics and sample preparation method, rather than strain specific effects, it may imply that probiotics affect the ratio between active and latent TGF-β in breast milk or the proportion of lipid bound TGF-β. Other factors are known to affect the proportion of active versus latent TGF-β, for example, mothers of premature infants having a higher proportion of latent TGF-β [30].

## Conclusions

Whilst maternal probiotic supplementation appears to result in higher breast milk concentrations of TLSP at 10 days postpartum, this does not seem contribute to the preventative effect of the probiotic regime on the development of AD. Probiotics did not significantly alter the breast milk concentration of any TGF-β isoforms at 10 days or 3 months postpartum. Further studies are required to both confirm these findings and investigate the other potential mechanism behind the prevention of AD in infancy by perinatal probiotic supplementation.

## Abbreviations

AD: atopic dermatitis
Bb12: *Bifidobacterium animalis* subsp. *lactis* Bb-12
ELISA: enzyme-linked immunospot assay
LGG: *Lactobacillus rhamnosus GG*
NDE: natural direct effect
NIE: natural indirect effect
OR: odds ratio
ProPACT: Probiotics in the Prevention of Allergy among Children in Trondheim
RR: risk ratio
TGF-β: transforming growth factor beta
Th2 cells: type 2 T helper cells
TSLP: thymic stromal lymphopoietin

## References

[1] Pelucchi C, Chatenoud L, Turati F, Galeone C, Moja L, Bach JF, La Vecchia C (2012). Probiotics supplementation during pregnancy or infancy for the prevention of atopic dermatitis: a meta-analysis. Epidemiology.

[2] Panduru M, Panduru NM, Salavastru CM, Tiplica GS (2015). Probiotics and primary prevention of atopic dermatitis: a meta-analysis of randomized controlled studies. J Eur Acad Dermatol Venereol JEADV.

[3] Dotterud CK, Storro O, Johnsen R, Oien T (2010). Probiotics in pregnant women to prevent allergic disease: a randomized, double-blind trial. Br J Dermatol.

[4] Dotterud CK, Avershina E, Sekelja M, Simpson MR, Rudi K, Storrø O, Johnsen R, Øien T (2015). Does maternal perinatal probiotic supplementation alter the intestinal microbiota of mother and child. J Pediat Gastroent Nutr.

[5] Hosea Blewett HJ, Cicalo MC, Holland CD, Field CJ, Steve LT (2008). Chapter 2—The immunological components of human milk. Advances in food and nutrition research.

[6] Ziegler SF (2012). Thymic stromal lymphopoietin and allergic disease. J Allergy Clin Immunol.

[7] Gao P-S, Rafaels NM, Mu D, Hand T, Murray T, Boguniewicz M, Hata T, Schneider L, Hanifin JM, Gallo RL (2010). Genetic variants in thymic stromal lymphopoietin are associated with atopic dermatitis and eczema herpeticum. J Allergy Clin Immunol.

[8] Kim J, Kim BE, Lee J, Han Y, Jun H-Y, Kim H, Choi J, Leung DYM, Ahn K (2016). Epidermal thymic stromal lymphopoietin predicts the development of atopic dermatitis during infancy. J Allergy Clin Immunol.

[9] Tatsuno K, Fujiyama T, Yamaguchi H, Waki M, Tokura Y (2015). TSLP directly interacts with skin-homing Th2 cells highly expressing its receptor to enhance IL-4 production in atopic dermatitis. J Invest Dermatol.

[10] Schwartz C, Eberle JU, Hoyler T, Diefenbach A, Lechmann M, Voehringer D. Opposing functions of TSLP-responsive basophils and dendritic cells in a mouse model of atopic dermatitis. J Allergy Clin Immunol. 2016. doi:10.1016/j.jaci.2016.04.031. 10.1016/j.jaci.2016.04.031 27372565

[11] Macfarlane TV, Seager AL, Moller M, Morgan G, Thornton CA (2010). Thymic stromal lymphopoietin is present in human breast milk. Pediatr Allergy Immunol.

[12] Nakao A (2010). The role and potential use of oral transforming growth factor-beta in the prevention of infant allergy. Clin Exp Allergy.

[13] Oddy WH, Rosales F (2010). A systematic review of the importance of milk TGF-beta on immunological outcomes in the infant and young child. Pediatr Allergy Immunol.

[14] Bottcher MF, Abrahamsson TR, Fredriksson M, Jakobsson T, Bjorksten B (2008). Low breast milk TGF-beta2 is induced by *Lactobacillus reuteri* supplementation and associates with reduced risk of sensitization during infancy. Pediatr Allergy Immunol.

[15] Kuitunen M, Kukkonen AK, Savilahti E (2012). Impact of maternal allergy and use of probiotics during pregnancy on breast milk cytokines and food antibodies and development of allergy in children until 5 years. Int Arch Allergy Immunol.

[16] Rautava S, Kalliomaki M, Isolauri E (2002). Probiotics during pregnancy and breast-feeding might confer immunomodulatory protection against atopic disease in the infant. J Allergy Clin Immunol.

[17] Huurre A, Laitinen K, Rautava S, Korkeamaki M, Isolauri E (2008). Impact of maternal atopy and probiotic supplementation during pregnancy on infant sensitization: a double-blind placebo-controlled study. Clin Exp Allergy.

[18] Prescott SL, Wickens K, Westcott L, Jung W, Currie H, Black PN, Stanley TV, Mitchell EA, Fitzharris P, Siebers R (2008). Supplementation with *Lactobacillus rhamnosus* or *Bifidobacterium lactis* probiotics in pregnancy increases cord blood interferon-gamma and breast milk transforming growth factor-beta and immunoglobin A detection. Clin Exp Allergy.

[19] Williams HC, Burney PG, Hay RJ, Archer CB, Shipley MJ, Hunter JJ, Bingham EA, Finlay AY, Pembroke AC, Graham-Brown RA (1994). The U.K. working party’s diagnostic criteria for atopic dermatitis. I. Derivation of a minimum set of discriminators for atopic dermatitis. Br J Dermatol.

[20] Simpson MR, Dotterud CK, Storrø O, Johnsen R, Øien T (2015). Perinatal probiotic supplementaion in the prevention of allergy related disease: 6 year follow up of a randomised controlled trial. BMC Dermatol.

[21] Valeri L, Vanderweele TJ (2013). Mediation analysis allowing for exposure-mediator interactions and causal interpretation: theoretical assumptions and implementation with SAS and SPSS macros. Psychol Methods.

[22] VanderWeele T (2015). Explanation in causal inference: methods for mediation and interaction.

[23] Liu H, Emsley R, Dunn G, VanderWeele T, Valeri L: PARAMED: Stata module to perform causal mediation analysis using parametric regression models. The Stata Journal 2014, Technical paper in preparation:Installation instructions for program code and help file available from: https://ideas.repec.org/c/boc/bocode/s457581.html.

[24] Vickery BP, Scurlock AM, Jones SM, Burks AW (2011). Mechanisms of immune tolerance relevant to food allergy. J Allergy Clin Immunol.

[25] Peterson LW, Artis D (2014). Intestinal epithelial cells: regulators of barrier function and immune homeostatis. Nat Rev Immunol.

[26] Ramirez-Santana C, Perez-Cano FJ, Audi C, Castell M, Moretones MG, Lopez-Sabater MC, Castellote C, Franch A (2012). Effects of cooling and freezing storage on the stability of bioactive factors in human colostrum. J Dairy Sci.

[27] Pontes GN, Cardoso EC, Carneiro-Sampaio MMS, Markus RP (2007). Pineal melatonin and the innate immune response: the TNF-alpha increase after cesarean section suppresses nocturnal melatonin production. J Pineal Res.

[28] Saarela T, Kokkonen J, Koivisto M (2005). Macronutrient and energy contents of human milk fractions during the first six months of lactation. Acta Paediatrica (Oslo, Norway: 1992).

[29] Filteau SM, Lietz G, Mulokozi G, Bilotta S, Henry CJK, Tomkins AM (1999). Milk cytokines and subclinical breast inflammation in Tanzanian women: effects of dietary red palm oil or sunflower oil supplementation. Immunology.

[30] Namachivayam K, Blanco CL, Frost BL, Reeves AA, Jagadeeswaran R, Mohankumar K, Safarulla A, Mandal P, Garzon SA, Raj JU (2013). Preterm human milk contains a large pool of latent TGF-beta, which can be activated by exogenous neuraminidase. Am J Physiol Gastrointest Liver Physiol.

